# Life: A Poem

**Published:** 1915-12

**Authors:** 


**Life: A Poem.** By BASIL GORDON MORISON, M.D. Pp. xv. 79. London: Baillière, Tindall and Cox. 1915. ]Price 3s. 6d. net.]

Dr. Morison, who died on January 28th, 1915, told his wife shortly before his death that "he had written a few verses which he thought might be considered worth publishing;" and some anonymous friend has edited these, with a Preface on "the Physician as Poet," and a biography.

The poem itself, composed in the metre of Gray's "Elegy," has very little that is new in form or thought; but it contains some of the opinions of an earnest man, expressed in carefully-considered, well-chosen words, and there is in it a spirit of pious resignation, hope and courage which might be taken to heart with advantage.

The Preface is well written, and the short biography gives us the picture of an energetic, useful and cultured man following his profession with a determination to do his best.

Some mysterious influence compels many men to try to express their thoughts and feelings in the form of verse. Whether it is wise for one who has no special gift for musical utterance to attempt this, when he could probably do it better in prose,is a question which cannot be discussed here. It may be said, however, that Dr. Morison's lines are as good as much that passes nowadays for poetry.

